# Physician and Practice Characteristics Influencing Telemedicine Uptake Among Frontline Clinicians in the Early COVID-19 Pandemic Response: National Survey Study

**DOI:** 10.2196/50751

**Published:** 2024-07-17

**Authors:** Michelle Y Hamline, Guibo Xing, Richard L Kravitz, Marykate Miller, Joy Melnikow

**Affiliations:** 1 Department of Pediatrics University of California Davis Sacramento, CA United States; 2 Center for Healthcare Policy and Research University of California Davis Sacramento, CA United States; 3 Department of Internal Medicine University of California Davis Sacramento, CA United States; 4 Department of Family and Community Medicine University of California Davis Sacramento, CA United States

**Keywords:** telemedicine, telehealth, COVID-19 pandemic, frontline clinicians, telemonitoring, frontliners, virtual care, influence, clinician, physician, pre-pandemic, pandemic, survey, health outcome

## Abstract

**Background:**

Telemedicine expanded rapidly during the COVID-19 pandemic, as key policy changes, financial support, and pandemic fears tipped the balance toward internet-based care. Despite this increased support and benefits to patients and clinicians, telemedicine uptake was variable across clinicians and practices. Little is known regarding physician and institutional characteristics underlying this variability.

**Objective:**

This study aimed to evaluate factors influencing telemedicine uptake among frontline physicians in the early pandemic response.

**Methods:**

We surveyed a national stratified sample of frontline clinicians drawn from the American Medical Association Physician Professional Data in June or July 2020. The survey inquired about the first month and most recent month (June 2020) of pandemic telemedicine use; sample data included clinician gender, specialty, census region, and years in practice. Local pandemic conditions were estimated from county-level data on COVID-19 rates at the time of survey response. Data were analyzed in a weighted logistic regression, controlling for county-specific pandemic data, and weighted to account for survey data stratification and nonresponse.

**Results:**

Over the first 3-4 months of the pandemic, the proportion of physicians reporting use of telemedicine in >30% of visits increased from 29.2% (70/239) to 35.7% (85/238). Relative to primary care, odds of substantial telemedicine use (>30%) both during the first month of the pandemic and in June 2020 were increased among infectious disease and critical care physicians and decreased among hospitalists and emergency medicine physicians. At least minimal prepandemic telemedicine use (odds ratio [OR] 11.41, 95% CI 1.34-97.04) and a high 2-week moving average of local COVID-19 cases (OR 10.16, 95% CI 2.07-49.97) were also associated with substantial telemedicine use in June 2020. There were no significant differences according to clinician gender, census region, or years in practice.

**Conclusions:**

Prepandemic telemedicine use, high local COVID-19 case counts, and clinician specialty were associated with higher levels of substantial telemedicine use during the early pandemic response. These results suggest that telemedicine uptake in the face of the pandemic may have been heavily influenced by the level of perceived threat and the resources available for implementation. Such understanding has important implications for reducing burnout and preparation for future public health emergencies.

## Introduction

Telemedicine expanded rapidly during the COVID-19 pandemic. Concerns about transmission fundamentally altered the relative risk versus benefit of telemedicine compared with in-person care [1-3]. Before the pandemic, telemedicine use was steadily rising, but adoption rates were low, as both patients and clinicians navigated complex logistical challenges and inadequate reimbursement [4]. However, one national cohort study found ambulatory contacts through telemedicine increased from 0.3% to 23.6%, in the first 3 months of the pandemic [5]. This transformation was supported by new legislation at the federal and state levels, implemented mostly on a temporary basis at the onset of the pandemic beginning in March 2020. This legislation provided payment parity for telemedicine visits, loosened restrictions on where telemedicine must originate and what platforms could be used, and provided critical funding to help boost telemedicine infrastructure [6].

Beyond the potential for improved safety from infectious transmission for both patients and clinicians, the benefits of telemedicine were demonstrated both before and during the pandemic. Patients report high satisfaction with telemedicine, citing convenience, ease of use, decreased costs, time savings, and a diversity of improved health outcomes, including improved quality of life and mental health [7-9]. Physicians across specialties similarly have developed favorable opinions of interacting with patients through telemedicine, noting comparable quality of care to in-person visits, cost-effectiveness, and time savings [10-13]. Physicians have also reported less burnout with telemedicine in comparison with in-person services, with increased flexibility and improved work-life balance [12,14,15]. Yet, despite this general satisfaction with telemedicine, some physicians and patients still favor in-person visits; this may be particularly true for specific patient complaints or visits requiring a physical exam [8,9,12].

The rapid expansion of telemedicine during the early pandemic provided a unique opportunity to appraise potential antecedents of and contributors to telemedicine adoption. Previous studies during the pandemic have established several factors influencing telemedicine uptake in an ambulatory setting, including higher use associated with increased COVID-19 prevalence in the patient’s area of residence during the preceding week and lower telemedicine use for patients living in areas with limited social resources [5]. Physicians have reported that over 75% of telemedicine visits occur with established patients, most often in a clinic setting [16]. Common uses reported for telemedicine include providing treatment, screening or diagnostics, and follow-up care [16].

Yet, despite the increased support for telemedicine associated with the pandemic and the documented benefits of telemedicine to both patients and clinicians, telemedicine uptake has remained variable across clinicians and practices. Little is known regarding physician and institutional characteristics underlying this variability, particularly for hospital-based settings [17]. We evaluated physician and institutional factors influencing telemedicine trends during the early pandemic response, through a survey of a national sample of inpatient and outpatient frontline clinicians early in the pandemic (June 2020), including previous telemedicine use (as an indicator of established telemedicine infrastructure); local COVID-19 pandemic trends within physician practice locations; and clinician gender, specialty, census region, and time in practice.

## Methods

### Population

The survey population and survey methods have been previously described in detail [18]. This study is reported according to the CHERRIES (Checklist for Reporting Results of Internet E-Surveys; Multimedia Appendix 1) [19]. Inclusion criteria included current US physicians within family practice, internal medicine, hospital medicine, critical care medicine, emergency medicine, and infectious disease. These specialties were selected to represent frontline physicians most likely to care for patients with acute COVID-19 infections. Early in the pandemic, it seemed COVID-19 infections were much less common and much less severe in children; hence, pediatricians were not included in the sample [20]. Specialties, such as obstetrics-gynecology and surgical specialties, that were less likely to be caring for patients with a chief complaint related to COVID-19 infection were considered outside the scope of this study.

A stratified national random sample of 10,000 US physicians was obtained from the American Medical Association (AMA) Physician Professional Data [21]. The AMA Physician Professional Data includes current and historical records for all physicians in the United States, including medical doctors (MDs) and osteopathic doctors (DOs). Formerly known as the AMA Physician Masterfile, it has been used in numerous studies of the physician workforce and physician surveys and found to be the best source of US physician sociodemographic and training information [22-26]. Obtaining a random sample from this comprehensive sampling frame ensured representativeness and provided data that enabled the analysis of both respondents and nonrespondents with weighting to address nonresponse bias. The sample was composed of 4000 primary care physicians (which included both family physicians and internists), 1000 hospitalists, 2000 critical care physicians (which included both critical care and pulmonary critical care physicians), 2000 emergency medicine physicians, and 1000 infectious disease physicians [21]. Hospitalists, intensivists, infectious disease physicians, and emergency medicine physicians were oversampled to help ensure adequate response rates. The survey was open only to this random sample of physicians. Because the response rate was unknown before fielding the survey, the sample size was estimated based on the assumption of a 10%-20% response rate [27].

### Survey

The survey questions were developed through a series of focus groups and interviews held in May 2020 with physicians from around the United States representing the included specialties. Draft survey questions were developed by the authors (JM and MM) based on key questions identified by these physicians in the focus groups and interviews. Several physicians in the representative specialties, including some who participated in the focus groups or interviews, reviewed the draft survey and provided feedback. The Qualtrics survey was distributed by email through a unique link to each participant in June or July 2020. The survey was distributed through email to the sample described above 3 times over a 3-week period to maximize response rates. The introductory email explained the purpose of the survey, and physicians could respond by clicking the link to start the survey. Participation was voluntary. The survey consisted of 48 questions over 15 pages, with adaptive questioning used to reduce the number and complexity of questions. A back button was available, but respondents were not able to review a summary of responses. Embedded data including a unique study ID were associated with each participant before distribution. These embedded data were automatically tied to each survey response, and duplicates were removed before analysis. IP addresses were not tracked. Responses were automatically captured by Qualtrics. Partially completed surveys were still included in the analysis.

The survey aimed to describe changes to practice during the early period of the COVID-19 pandemic. Primary outcomes were the impact of the pandemic on patient care and practice structure, with a major focus on the adoption of telemedicine. We collected data on factors that might affect telemedicine implementation, including type of practice and confirming specialty. Gender, years in practice, and zip code were provided on the sample by the AMA Physician Professional Data. The survey asked about prepandemic telemedicine use rates and how these changed during the initial period of the pandemic and specifically inquired about telemedicine use rates within the first month of the pandemic and the month preceding survey completion (June 2020). The first month of the pandemic was defined as the first month of the pandemic in the physician’s local area, so varied based on individual physician location. In addition, free text responses were allowed in response to questions inquiring about how telemedicine had impacted physicians’ practice during the pandemic and the most important impacts the pandemic has had on the physicians’ practice.

### Analysis

Consistent with standard survey methodology, surveys returned due to an invalid email were removed from the denominator, as it could not be determined whether the email truly belonged to a person who should have been included in the sampling frame [28]. Responses were weighted to improve their representativeness and to reduce nonresponse bias as previously described [18]. Because some smaller specialties were oversampled to ensure representation, survey design weights were constructed as the inverse probability of selection into the sample. Entropy balancing, a nonparametric generalization of propensity score weighting, was applied to address nonresponse bias [29]. The nonresponse weights were constructed such that the mean, variance, and skewness of the DOs, females, years in practice, and type of practice among respondents matched the full stratified sample.

Data were analyzed in a weighted logistic regression to identify factors associated with the dependent variable: higher or lower odds of telemedicine use in the first month of the pandemic (for the respondent’s region) and in June 2020. Independent variables were included in the analysis based on the research team’s assessment (informed by physician focus groups and interviews) that they might be influential in the level of telemedicine adoption. For those with missing values for telemedicine use during the pandemic, if the respondent answered the first question of the telemedicine section of the survey and continued to complete the first question in the survey following the telemedicine section, we assumed that telemedicine use had remained unchanged and utilized prepandemic telemedicine use. To adjust for local pandemic-related confounding variation in the intensity of the COVID-19 pandemic, we linked each physician’s city and state of residence from the AMA Physician Professional Data to counties. County-level daily COVID-19 case count data were obtained from the COVID-19 Data Repository maintained by the Center for Systems Science and Engineering at Johns Hopkins University. SURVEYLOGISTIC procedure in SAS (SAS Institute) was used for weighted multivariate logistic regressions; SAS version 9.4 and Stata/MP 16 (StataCorp LLC) were used for data processing and analyses.

### Ethical Considerations

This study was reviewed by the University of California Davis Institutional Review Board and met the criteria for exemption (IRB ID 1593608). Therefore, informed consent was obtained through respondent review of an email cover letter containing the anticipated survey length, primary investigator contact information, study purpose, and the link to the physician survey. To protect participant privacy, data were collected anonymously, but physicians were offered the opportunity to provide contact information to be included in a lottery for a gift card or to participate in future studies. No other participant compensation was provided.

## Results

The survey yielded 286 responses. After eliminating 1285 undelivered emails, the final denominator was 8715, for a 3.3% response rate. This is within the range reported for several previously published physician surveys using the AMA Physician Professional Data [25,30,31]. Based on data for the total sample (including nonresponders) from the AMA Physician Professional Data, responses were weighted to represent the sampled population. After weighting, survey responders were similar to nonresponders according to known characteristics including type of medical training (MD vs DO), gender, physician specialty, years in practice, type of practice, and census region [18]. Weighted balance statistics indicated near-perfect balance. Descriptive statistics comparing responders versus nonresponders after weighting are shown in Table 1.

Most respondents (263/286 92.0%) reported using telemedicine for less than 10% of patients before the pandemic. Telemedicine use rose rapidly early in the pandemic, with 29.2% (70/239) and 35.7% (85/238) of physicians reporting using telemedicine for at least 30% of patients in the first month of the pandemic (in their region) and in June 2020, respectively (data not shown in tabular form). After examining the descriptive data, we focused on cutoff points of 10% and 30% of patient visits being performed with telemedicine, which we defined as at least minimal and substantial telemedicine use, respectively.

We next analyzed the proportion of physicians reporting substantial telemedicine use in the prepandemic time period, the first month of the pandemic, and June 2020, according to physician specialty and years in practice. In the first month of the pandemic, hospitalists (4/34, 11.8%) and emergency medicine physicians (2/42, 4.8%) were least likely to be using telemedicine substantially, while primary care providers (34/72, 47.2%), infectious disease physicians (12/29, 41.4%), and critical care physicians (18/62, 29.0%) were more likely to be using telemedicine substantially. By June 2020, the proportion of physicians reporting substantial telemedicine use had increased for all specialties except hospitalists (3/34, 8.8%), with 11.9% (5/42) of emergency medicine physicians, 52.8% (38/72) of primary care physicians, 65.5% (19/29) of infectious disease physicians, and 32.8% (20/61) of critical care physicians reporting substantial use. However, in June 2020, both hospitalists and emergency medicine physicians remained less likely to use telemedicine substantially than their peers in primary care.

Substantial telemedicine use also varied by years in practice. In the first month of the pandemic, physicians practicing for 0-30 years were most likely to use telemedicine substantially, with substantial use reported by 26.4% (19/72) of those in practice for 0-10 years, 26.0% (20/77) of those practicing for 11-20 years, and 41.1% (23/56) of physicians in practice for 21-30 years. Lowest telemedicine use in the first month of the pandemic was seen in physicians practicing for over 30 years, with 24.0% (6/25) of physicians in practice for 31-40 years and 12.5% (1/8) of physicians in practice more than 40 years reporting substantial use. By June 2020, telemedicine use increased for all groups of physicians with over 10 years in practice, most notably those with more than 40 years in practice (11-20 years: 33.8% [26/77], 21-30 years 47.3% [26/55], 31-40 years 40.0% [10/25], more than 40 years 37.5% [3/8]).

Logistic regression (Table 2) revealed at least minimal telemedicine use prepandemic was not significantly associated with substantial telemedicine use in the first month of the pandemic. In contrast, substantial telemedicine use in June 2020 was strongly associated with at least minimal telemedicine use prepandemic (odds ratio [OR] 11.41, 95% CI 1.34-97.04). By June 2020, regional pandemic conditions, represented by the 2-week moving average of local cases, also had a significant impact on substantial telemedicine use (OR 10.16, 95% CI 2.07-49.97). Clinician specialty was significantly correlated with substantial telemedicine use. Both within the first month of the pandemic and in June 2020, hospitalists (first month: OR 0.14, 95% CI 0.03-0.65; June 2020: OR 0.05, 95% CI 0.01-0.26) and emergency medicine physicians (first month: OR 0.05, 95% CI 0.01-0.36; June 2020: OR 0.064, 95% CI 0.01-0.32) were least likely to be using telemedicine substantially, while primary care providers (reference), infectious disease physicians, and critical care physicians were more likely to be using telemedicine substantially. Regional differences in telemedicine use showed greater odds ratios of use in the Northeast and West compared with the Midwest by June 2020, but these differences were not statistically significant. Other clinician characteristics, including gender and years in practice, were not significantly associated with greater use of telemedicine at either time point.

**Table 1:** Physician and practice characteristics of survey respondents versus nonrespondents.

Characteristics		Response status				Weighted balance^a^	
		Did not respond	Responded				
		n (%)	n (%)	Weighted% (mean)	Mean^b^		Ratio^c^
**All**		8429 (96.7)	286 (3.3)				
**Medical training**							
	MD^d^	7811 (92.7)	269 (94.1)	92.8			
	DO^e^	618 (7.3)	17 (5.9)	7.2	0.000		1.003
**Sex**							
	Female	2812 (33.4)	121 (42.3)	33.7	0.000		1.003
	Male	5617 (66.6)	165 (57.7)	66.3			
**Physician specialty**							
	Critical care medicine	838 (9.9)	42 (14.7)	10.1			
	Emergency medicine	1627 (19.3)	51 (17.8)	19.3	0.000		1.003
	Family medicine	1719 (20.4)	48 (16.8)	20.2	0.000		1.003
	Hospitalist	848 (10.1)	41 (14.3)	10.2	0.000		1.003
	Infectious disease	838 (9.9)	32 (11.2)	10	0.000		1.003
	Internal medicine	1710 (20.3)	42 (14.7)	20.1	0.000		1.003
	Pulmonary critical care medicine	849 (10.1)	29 (10.1)	10.1	0.000		1.003
**Type of practice**							
	Office	6114 (72.5)	198 (69.2)	72.4			
	Hospital staff	2181 (25.9)	81 (28.3)	26	0.000		1.003
	Teaching	134 (1.6)	6 (2.1)	1.6	0.000		1.003
Years since residency^f^		17.8 (11)	18.1 (10.7)	17.8	0.000		1.003

^a^Weighted balance is based on diagnostic output produced by the kmatch module.

^b^Mean is the standard difference in means between weighted respondents and weighted nonrespondents; standard difference is 0 when perfectly balanced. Standard difference in means is rounded to 3 significant digits.

^c^Ratio represents the ratio of variances of weighted nonrespondents to variance of weighted respondents. Ratio is 1 when perfectly balanced. Ratio of variances is rounded to 3 significant digits.

^d^MD: medical doctor.

^e^DO: osteopathic doctor.

^f^Reports mean years since residency.

**Table 2:** Multivariable logistic regression analysis showing the association between substantial telemedicine use in the first local month of the pandemic and June 2020 with local pandemic conditions, practice, and provider characteristics. Substantial use is defined as telemedicine use for at least 30% of patients.

Characteristics		Substantial first-month pandemic telemedicine use, OR^a^ (95% CI)	Substantial telemedicine use June 2020, OR (95% CI)		
**Prepandemic telemedicine use**					
	<10% of patients	Ref^b^	Ref		
	>10% of patients	2.65 (0.37-18.80)	*11.41 (1.34-97.04)* ^c^		
**2-week moving average of local COVID-19 cases**					
	Low	Ref	Ref		
	High	5.21 (0.96-28.35)	*10.16 (2.07-49.97)*		
**Provider census region**					
	Midwest	Ref	Ref		
	Northeast	1.92 (0.45-8.14)	3.3 (0.90-12.05)		
	South	0.53 (0.10-2.84)	1.19 (0.24-5.95)		
	West	1.10 (0.33-3.67)	2.81 (0.88-9.01)		
**Provider sex**					
	Female	Ref	Ref		
	Male	0.57 (0.22-1.49)	0.73 (0.27-1.93)		
**Provider specialty**					
	Primary care	Ref	Ref		
	Critical care	1.57 (0.51-4.84)	0.90 (0.28-2.90)		
	Emergency medicine	*0.05 (0.01-0.36)*	*0.06 (0.01-0.32)*		
	Hospital medicine	*0.14 (0.03-0.65)*	*0.05 (0.01-0.26)*		
	Infectious disease	0.95 (0.32-2.85)	2.27 (0.71-7.30)		
**Provider years in practice**					
	0-10 years	Ref	Ref		
	11-20 years	1.87 (0.50-6.98)	1.90 (0.54-6.60)		
	21-30 years	1.92 (0.60-6.15)	1.97 (0.55-7.03)		
	31-40 years	1.11 (0.22-5.63)	1.12 (0.20-6.29)		
	More than 40 years	0.29 (0.02-3.91)	1.27 (0.15-10.69)		

^a^OR: odds ratio.

^b^Ref: reference.

^c^Italicized values are significant.

## Discussion

### Principal Findings

This cross-sectional national survey of frontline clinicians found that higher rates of telemedicine adoption by early June 2020 were associated with prepandemic telemedicine use and recent local COVID-19 case counts. This is the first study that we know of to compare pandemic telemedicine use across outpatient and inpatient frontline specialties. Increases in the use of telemedicine were noted in all frontline specialties but were most marked for infectious disease, critical care, and primary care physicians.

### Comparison With Previous Work

Across physician gender, specialties, census regions, and years in practice, this study found a substantial increase in telemedicine use in the early months of the pandemic. Previous studies have shown similar rapid telemedicine uptake, but these studies have focused primarily on clinic-based specialties [5,6,32,33]. This study shows telemedicine use rates increased with similar rapidity in the hospital-based specialty of critical care, with less substantial increases seen also in emergency and hospital medicine. Telemedicine use continued to rise through 2021, as pandemic fears persisted and telemedicine infrastructure continued to expand [16].

The increased use of telemedicine during the pandemic among those who had previously used telemedicine is not surprising, as this likely reflects preexisting local infrastructure for telemedicine. Previous use of telemedicine implies hospital and physician readiness to ramp up the use. Multiple previous telemedicine implementation models have emphasized the necessity of preexisting infrastructure, including coordinated hardware and software platforms, audiovisual integration, 24/7 information technology support, and clinician training in achieving telemedicine success [34-36]. Previous work has shown that successful completion of telemedicine relies, in part, on clinician comfort with technology [37].

If we consider telemedicine as a preventative strategy in the face of the pandemic threat to patient and clinician safety, our findings are consistent with the Protection Motivation Theory (PMT), a behavioral theory developed to understand human responses to fear [38]. PMT posits that response to fear-inducing situations is influenced by 2 main factors that are (1) threat appraisal (an individual’s perceived severity of and vulnerability to the threat), and (2) coping appraisal (an individual’s ability to respond to the threat with resources at hand). Applied to this study, telemedicine uptake may be influenced by an individual’s perceived threat from the pandemic, as well as their belief (or lack of belief) that telemedicine will help them respond to that threat, which is likely dependent on both environmental and individual factors. Furthermore, exploration through qualitative analyses may help more clearly explain how PMT factors of threat and coping appraisal impact telemedicine uptake and other adaptations in the face of pandemic threat. Such exploration may have important implications for the adoption of new technologies in responding to future public health emergencies.

It is notable that primary care, infectious disease, and critical care physicians reported higher pandemic telemedicine use than hospitalists and emergency medicine physicians. While several previous studies have evaluated telemedicine use across various outpatient specialties [16,33,39], in free text responses to our survey, several physicians reported that their hospital did not yet have the infrastructure to conduct telemedicine outside of the clinic setting, which could in part explain the higher rates of use among primary care and infectious disease physicians. However, an additional explanatory factor may lie within PMT, as previous studies have suggested primary care and critical care physicians were at the highest risk of contracting and dying from COVID-19 infection [40,41]. Notably, we found higher telemedicine adoption by physicians in regions where the 2-week moving average of COVID-19 cases was high, a situation that increased real and perceived threat, as well as an early rise in telemedicine adoption in the Northeast, the region that experienced pandemic surges before nationwide spread.

It is also notable that several studies have previously reported that telemedicine helps reduce physician burnout [12,14,15]. Therefore, our findings regarding factors influencing telemedicine uptake may have important implications for reducing physician burnout, which has known associations with physician turnover, mental health, and medical error [25,42].

Within the hospital setting, participants’ free text responses noted that telemedicine was often used for remote consultation and family communication. For example, one participant noted that telemedicine allowed for “improved communication with the family diaspora.” Consistent with our findings, another survey study of critical care physicians during the pandemic reported telemedicine was most frequently used in intensive care unit settings for clinician, nurse, and patient communication with patient families [43]. These communications varied from general updates on patient conditions to more in-depth goals of care discussions.

### Limitations

This study was limited by low survey response rates and the potential for selection bias. Our ability to weight responses based on known characteristics of the total sample minimized nonresponse bias, but there is the possibility of enduring bias in unmeasured characteristics. The number of respondents limited our ability to assess differences based on respondent characteristics. Another potential limitation is that of coverage bias, particularly with respect to the undeliverable surveys due to bounced emails. The characteristics of these individuals were unknown, including whether they were still in practice, and these units were therefore eliminated from the study sample, as is standard practice [28].

We did not explicitly collect information regarding prepandemic technology readiness but rather used prepandemic telemedicine as a proxy. Therefore, we cannot draw an explicit association, but rather can only infer that prepandemic technology readiness may have impacted pandemic telemedicine use. It is also possible that patient characteristics and preferences drove telemedicine uptake during the pandemic, but these factors were not evaluated in this study. Finally, since this study focused on the use of telemedicine in the early pandemic response for only a subset of specialties, we cannot provide information regarding telemedicine use in other specialties or regarding the longevity of telemedicine use throughout the pandemic, although other published works have addressed later time points [16].

### Future Work

The most important finding of this study was the capacity for rapid uptake of telemedicine under the right set of conditions—in particular, a preexisting telemedicine infrastructure combined with improved reimbursement and clear evidence of benefit given pandemic risks. Health care is notoriously slow to incorporate innovative, evidence-based technologies. However, our data show that telemedicine uptake in the early pandemic was rapid across genders, specialties, geographic regions, and experience levels. Rogers’ theory of diffusion of innovation posits a 5-step process involving knowledge, persuasion, decision, implementation, and confirmation [44]. This process puts the adopter (in this case, often the clinician) at the center of implementation. However, the pandemic-era implementation of telemedicine highlights the critical role of other factors, such as public policy, external supports, health system or practice and patient infrastructure, and customer demand, in the widespread adoption of a new technology.

Telemedicine use is at a critical juncture as the public health emergency has expired. Clinicians and patients alike have shared its benefits and have developed increasing comfort levels with telemedicine technology; studies during the pandemic showed that patients who received telemedicine visits had higher average patient satisfaction scores than those seen in-person [45]. However, recent studies suggest that both patients and physicians tend to prefer in-person care, seeking to move away from telemedicine in the postpandemic era [46]. Still, telemedicine may be particularly well suited to specific patient care scenarios, such as ongoing medical management of chronic conditions, behavioral health care, and communicating with families about hospitalized patients [9]. Future research must determine optimal applications of telemedicine within each specialty, and what factors will drive its continued use as pandemic fears recede and threat appraisal dissipates.

Continued use of telemedicine will also depend largely on enduring compensation policies. On November 1, 2022, the Centers for Medicare and Medicaid Services issued the 2023 Physician Fee Schedule Final Rule, which began peeling back some of the temporary telemedicine allowances passed in affiliation with the COVID-19 public health emergency. A summary of pandemic-era telemedicine policy changes with actual and impending end dates is provided in Table 3 [47-51].

**Table 3:** Pandemic-era telemedicine policy changes with actual and anticipated end dates.

Pandemic-era telemedicine policy changes	Actual and anticipated end dates
Suspension of HIPAA^a^ restrictions on allowable telemedicine platforms	May 11, 2023
Temporary payment parity rules for telemedicine visits	December 31, 2023
Compensation for audio-only telephone evaluation and management services	December 31, 2024
Internet-based direct supervision of health care services	December 31, 2024
Suspension of geographic and originating site restrictions for nonbehavioral telemedicine services	December 31, 2024
Temporary telemedicine billing codes, such as those for hospital-based telemedicine encounters	December 31, 2024

^a^Health Insurance Portability and Accountability Act.

The potential implications of these looming expirations are far-reaching given the widespread telemedicine uptake across specialties demonstrated in our study and others. The inability to bill for critical care telemedicine, which is largely leveraged to involve remote family members in decision-making and care coordination [43], could impact the ability to provide patient- and family-centered care. Elimination of direct online supervision will dramatically reduce the exposure of trainees to the practice of telemedicine. Loss of compensation for telephone visits will reduce access to care for low-income and rural patients who have less access to broadband internet. Meanwhile, the reinstatement of geographic and originating site restrictions, and the expiration of payment parity rules, could drastically limit telemedicine encounters even in the outpatient setting, resulting in a large-scale reduction in telemedicine use across specialties compared with pandemic levels. While many states have implemented policies requiring payment parity from private payors, other states have not yet implemented such requirements, and these requirements do not apply to Medicaid [52]. A final consideration in the roll-back of policies supporting telemedicine is the potential effect on readiness for the next pandemic. Maintaining support and infrastructure for telemedicine may enable rapid and life-saving transitions to remote care.

### Conclusion

The COVID-19 pandemic brought about rapid incorporation of telemedicine across health care. This survey of frontline clinicians found higher rates of telemedicine adoption in response to the pandemic for physicians working in counties with higher COVID-19 case rates and for physicians with higher prepandemic telemedicine use, particularly in primary care, infectious disease, and critical care specialties. These findings have important implications for the ongoing adoption and maintenance of telemedicine to help reduce burnout, as well as key lessons for responding to public health emergencies. Future research must evaluate the use of telemedicine compared with in-person care on health outcomes and address the impact of policy changes on continued telemedicine use. To sustain the use of telemedicine across settings, the potential benefits of telemedicine in providing patient- and family-centered care and the importance of trainee experience in telemedicine must be communicated to policymakers and the public.

## Appendix

Multimedia Appendix 1 CHERRIES (Checklist for Reporting Results of Internet E-Surveys).

## Abbreviations

AMA: American Medical Association
CHERRIES: Checklist for Reporting Results of Internet E-Surveys
DO: osteopathic doctor
MD: medical doctor
OR: odds ratio
PMT: Protection Motivation Theory

## Data Availability

The data sets generated or analyzed during this study are available from the corresponding author on reasonable request.

## References

[1] Mann DM, Chen J, Chunara R, Testa PA, Nov O (2020). Covid-19 transforms health care through telemedicine: evidence from the field. J Am Med Inform Assoc.

[2] Mohammed HT, Hyseni L, Bui V, Gerritsen B, Fuller K, Sung J, Alarakhia M (2021). Exploring the use and challenges of implementing virtual visits during Covid-19 in primary care and lessons for sustained use. PLoS One.

[3] Hatef E, Wilson RF, Hannum SM, Zhang A, Kharrazi H, Weiner JP, Davis SA, Robinson KA (2023). Use of telehealth during the COVID-19 era [internet].

[4] Weigel G (2020). Womens Health Policy: opportunities and barriers for telemedicine in the U.S. during the COVID-19 emergency and beyond.

[5] Weiner JP, Bandeian S, Hatef E, Lans D, Liu A, Lemke KW (2021). In-person and telehealth ambulatory contacts and costs in a large US insured cohort before and during the COVID-19 pandemic. JAMA Netw Open.

[6] Shaver J (2022). The state of telehealth before and after the COVID-19 pandemic. Prim Care.

[7] Kruse CS, Krowski N, Rodriguez B, Tran L, Vela J, Brooks M (2017). Telehealth and patient satisfaction: a systematic review and narrative analysis. BMJ Open.

[8] Polinski JM, Barker T, Gagliano N, Sussman A, Brennan TA, Shrank WH (2016). Patients' satisfaction with and preference for telehealth visits. J Gen Intern Med.

[9] Orlando JF, Beard M, Kumar S (2019). Systematic review of patient and caregivers' satisfaction with telehealth videoconferencing as a mode of service delivery in managing patients' health. PLoS One.

[10] Alkureishi MA, Choo Z, Lenti G, Castaneda J, Zhu M, Nunes K, Weyer G, Oyler J, Shah S, Lee WW (2021). Clinician perspectives on telemedicine: observational cross-sectional study. JMIR Hum Factors.

[11] Gold KJ, Laurie AR, Kinney DR, Harmes KM, Serlin DC (2021). Video visits: family physician experiences with uptake during the COVID-19 pandemic. Fam Med.

[12] Malouff TD, TerKonda SP, Knight D, Abu Dabrh AM, Perlman AI, Munipalli B, Dudenkov DV, Heckman MG, White LJ, Wert KM, Pascual JM, Rivera FA, Shoaei MM, Leak MA, Harrell AC, Trifiletti DM, Buskirk SJ (2021). Physician satisfaction with telemedicine during the COVID-19 pandemic: the Mayo Clinic Florida experience. Mayo Clin Proc Innov Qual Outcomes.

[13] Naqvi SZ, Ahmad S, Rocha IC, Ramos KG, Javed H, Yasin F, Khan HD, Farid S, Mohsin A, Idrees A (2022). Healthcare workers' knowledge and attitude toward telemedicine during the COVID-19 pandemic: a global survey. Cureus.

[14] Brault ME, Laudermith A, Kroll-Desrosiers A (2023). Telemedicine during COVID-19 response: a welcome shift for younger female healthcare workers. J Gen Intern Med.

[15] Mills K, Peterson A, McNair M, Abe T, Igwe J, Sobukonla T, Surapaneni P, Ajose T, Yan F, Chang E, Volcy J (2022). Virtually serving the underserved: resident perceptions of telemedicine use while training during coronavirus disease 2019. Telemed J E Health.

[16] (2021). Telehealth survey report. American Medical Association.

[17] Robeznieks A (2022). Inside the big variations in telehealth use among physicians. AMA Recovery Plan for Physicians.

[18] Melnikow J, Padovani A, Miller M (2022). Frontline physician burnout during the COVID-19 pandemic: national survey findings. BMC Health Serv Res.

[19] Eysenbach G (2004). Improving the quality of web surveys: the Checklist for Reporting Results of Internet e-Surveys (CHERRIES). J Med Internet Res.

[20] Swann OV, Holden KA, Turtle L, Pollock L, Fairfield CJ, Drake TM, Seth S, Egan C, Hardwick HE, Halpin S, Girvan M, Donohue C, Pritchard M, Patel LB, Ladhani S, Sigfrid L, Sinha IP, Olliaro PL, Nguyen-Van-Tam JS, Horby PW, Merson L, Carson G, Dunning J, Openshaw PJM, Baillie JK, Harrison EM, Docherty AB, Semple MG, ISARIC4C Investigators (2020). Clinical characteristics of children and young people admitted to hospital with COVID-19 in United Kingdom: prospective multicentre observational cohort study. BMJ.

[21] (2023). AMA Physician Professional Data. American Medical Association.

[22] U.S. Physician Workforce Data Dashboard. Association of American Medical Colleges.

[23] Robboy SJ, Gross D, Park JY, Kittrie E, Crawford JM, Johnson RL, Cohen MB, Karcher DS, Hoffman RD, Smith AT, Black-Schaffer WS (2020). Reevaluation of the US Pathologist workforce size. JAMA Netw Open.

[24] Elkbuli A, Sutherland M, Sanchez C, Liu H, Ang D, McKenney M (2022). The shortage of trauma surgeons in the US. Am Surg.

[25] Menon NK, Shanafelt TD, Sinsky CA, Linzer M, Carlasare L, Brady KJS, Stillman MJ, Trockel MT (2020). Association of physician burnout with suicidal ideation and medical errors. JAMA Netw Open.

[26] Baldwin L, Adamache W, Klabunde CN, Kenward K, Dahlman C, Warren JL (2002). Linking physician characteristics and medicare claims data: issues in data availability, quality, and measurement. Med Care.

[27] Dillman D, Smyth J, Christian L (2014). Internet, phone, mail, and mixed-mode surveys: the tailored design method.

[28] Valliant R, Dever J, Kreuter F (2013). Practical tools for designing and weighting survey samples.

[29] Hainmueller J (2017). Entropy balancing for causal effects: A multivariate reweighting method to produce balanced samples in observational studies. Political Analysis.

[30] Murphy CC, Craddock Lee SJ, Geiger AM, Cox JV, Ahn C, Nair R, Gerber DE, Halm EA, McCallister K, Skinner CS (2020). A randomized trial of mail and email recruitment strategies for a physician survey on clinical trial accrual. BMC Med Res Methodol.

[31] Nair D, Brereton L, Hoge C, Plantinga LC, Agrawal V, Soman SS, Choi MJ, Jaar BG, National Kidney Foundation Education Committee (2022). Burnout among nephrologists in the United States: a survey study. Kidney Med.

[32] Pierce BS, Perrin PB, Dow AW, Dautovich ND, Rybarczyk BD, Mishra VK (2021). Changes in physician telemedicine use during COVID-19: effects of practice setting, demographics, training, and organizational policies. Int J Environ Res Public Health.

[33] Hsiao V, Chandereng T, Huebner JA, Kunstman DT, Flood GE, Tevaarwerk AJ, Schneider DF (2023). Telemedicine use across medical specialties and diagnoses. Appl Clin Inform.

[34] Meyer BC, Friedman LS, Payne K, Moore L, Cressler J, Holberg S, Partridge B, Prince B, Sylwestrzak M, Jenusaitis M, Kremer B, Kane CJ, Sitapati A, Clay B, Millen M, Longhurst C (2021). Medical undistancing through telemedicine: a model enabling rapid telemedicine deployment in an academic health center during the Covid-19 pandemic. Telemed J E Health.

[35] Smith WR, Atala AJ, Terlecki RP, Kelly EE, Matthews CA (2020). Implementation guide for rapid integration of an outpatient telemedicine program during the COVID-19 pandemic. J Am Coll Surg.

[36] Kreofsky BLH, Blegen RN, Lokken TG, Kapraun SM, Bushman MS, Demaerschalk BM (2018). Sustainable telemedicine: designing and building infrastructure to support a comprehensive telemedicine practice. Telemed J E Health.

[37] Crotty BH, Hyun N, Polovneff A, Dong Y, Decker MC, Mortensen N, Holt JM, Winn AN, Laud PW, Somai MM (2021). Analysis of clinician and patient factors and completion of telemedicine appointments using video. JAMA Netw Open.

[38] Rogers RW (1975). A protection motivation theory of fear appeals and attitude change. J Psychol.

[39] Drake C, Lian T, Cameron B, Medynskaya K, Bosworth HB, Shah K (2022). Understanding telemedicine's "new normal": variations in telemedicine use by specialty line and patient demographics. Telemed J E Health.

[40] Gouda D, Singh PM, Gouda P, Goudra B (2021). An overview of health care worker reported deaths during the COVID-19 pandemic. J Am Board Fam Med.

[41] Bandyopadhyay S, Baticulon RE, Kadhum M, Alser M, Ojuka DK, Badereddin Y, Kamath A, Parepalli SA, Brown G, Iharchane S, Gandino S, Markovic-Obiago Z, Scott S, Manirambona E, Machhada A, Aggarwal A, Benazaize L, Ibrahim M, Kim D, Tol I, Taylor EH, Knighton A, Bbaale D, Jasim D, Alghoul H, Reddy H, Abuelgasim H, Saini K, Sigler A, Abuelgasim L, Moran-Romero M, Kumarendran M, Jamie NA, Ali O, Sudarshan R, Dean R, Kissyova R, Kelzang S, Roche S, Ahsan T, Mohamed Y, Dube AM, Gwini GP, Gwokyala R, Brown R, Papon MRKK, Li Z, Ruzats SS, Charuvila S, Peter N, Khalidy K, Moyo N, Alser O, Solano A, Robles-Perez E, Tariq A, Gaddah M, Kolovos S, Muchemwa FC, Saleh A, Gosman A, Pinedo-Villanueva R, Jani A, Khundkar R (2020). Infection and mortality of healthcare workers worldwide from COVID-19: a systematic review. BMJ Glob Health.

[42] Lagasse J (2022). Cost of burnout-related physician turnover totals $5 billion annually. Healthcare Finance.

[43] Nelson SE, Steuernagle J, Rotello L, Nyquist P, Suarez JI, Ziai W (2022). COVID-19 and telehealth in the intensive care unit setting: a survey. BMC Health Serv Res.

[44] Rogers EM (2003). Diffusion of Innovations.

[45] Ramaswamy A, Yu M, Drangsholt S, Ng E, Culligan PJ, Schlegel PN, Hu JC (2020). Patient satisfaction with telemedicine during the COVID-19 pandemic: retrospective cohort study. J Med Internet Res.

[46] SteelFisher GK, McMurtry CL, Caporello H, Lubell KM, Koonin LM, Neri AJ, Ben-Porath EN, Mehrotra A, McGowan E, Espino LC, Barnett ML (2023). Video telemedicine experiences in COVID-19 were positive, but physicians and patients prefer in-person care for the future. Health Aff (Millwood).

[47] Morse S (2023). Telehealth payment parity only good through 2023.

[48] Fact sheet 2022. CY 2023 Medicare Hospital Outpatient Prospective Payment System and Ambulatory Surgical Center Payment System Final Rule with Comment Period (CMS 1772-FC).

[49] (2023). H.R.2617 - Consolidated Appropriations Act.

[50] Telehealth policy changes after the COVID-19 public health emergency.

[51] (2022). 2023 Physician Fee Schedule Final Rule.

[52] (2023). Billing for providers: what should I know?.

